# Methods for measuring egocentric distance perception in visual modality

**DOI:** 10.3389/fpsyg.2022.1061917

**Published:** 2023-01-11

**Authors:** Bo Dong, Airui Chen, Zhengyin Gu, Yuan Sun, Xiuling Zhang, Xiaoming Tian

**Affiliations:** ^1^Department of Psychology, Suzhou University of Science and Technology, Suzhou, China; ^2^Department of Psychology, Zhejiang Sci-Tech University, Hangzhou, China; ^3^School of Education, Suzhou University of Science and Technology, Suzhou, China; ^4^School of Psychology, Northeast Normal University, Changchun, China

**Keywords:** egocentric distance perception, spatial perception, measurement methods, perceptual method, direct action method, indirect action method

## Abstract

Egocentric distance perception has been widely concerned by researchers in the field of spatial perception due to its significance in daily life. The frame of perception involves the perceived distance from an observer to an object. Over the years, researchers have been searching for an optimal way to measure the perceived distance and their contribution constitutes a critical aspect of the field. This paper summarizes the methodological findings and divides the measurement methods for egocentric distance perception into three categories according to the behavior types. The first is Perceptional Method, including successive equal-appearing intervals of distance judgment measurement, verbal report, and perceptual distance matching task. The second is Directed **Action** Method, including blind walking, blind-walking gesturing, blindfolded throwing, and blind rope pulling. The last one is Indirect **Action** Method, including triangulation-by-pointing and triangulation-by-walking. In the meantime, we summarize each method’s procedure, core logic, scope of application, advantages, and disadvantages. In the end, we discuss the future concerns of egocentric distance perception.

## 1. Introduction

Space is the basis of the interaction between human beings and the environment. “How do human beings perceive space?” is a classical problem in cognitive psychology and ecological psychology. With the emergence of interdisciplinary studies, the problem constitutes the foundation of environmental psychology. The “mystery” of spatial perception is that the visual system can reproduce three-dimensional space depending on two-dimensional retinal images (Gibson, 1950). The transformation mentioned above can be called depth perception. Specifically, depth perception includes exocentric distance perception, which is about the distance from one object to another, and egocentric distance perception, which is about the distance from an observer to an object (Blake and Sekuler, 2006; Ooi and He, 2007). Egocentric distance perception supports humans in representing the location of objects and constructing the visual space of the environment (Gibson, 1950; Loomis et al., 1996). When humans take action in daily life, for example, taking a cup, driving a car, or throwing a draft, egocentric distance perception plays an essential role in the procedure. To some extent, egocentric distance perception is a precondition for survival.

According to the relative motion state between the observer and the object, egocentric distance perception can be divided into static state and dynamic state (Blake and Sekuler, 2006). For the static state, the observer and the target keep still, and for the dynamic state, the target or the observer is in motion. Egocentric distance perception in the dynamic state involves complex cognitive procedures, for example, motion parallax, high-speed spatial updating, speed perception, and ontological motion. Due to the complexity, there were few studies about it, and the study of its internal mechanism was still in infancy (Santillán and Barraza, 2019; Dong et al., 2021a). By contrast, researchers have studied egocentric distance perception in the static state more profoundly, and more than 10 methods have been developed, which have been widely used in laboratory research and business development. Furthermore, in essence, static distance perception is the basis and premise of dynamic distance perception, so the following will focus on egocentric distance perception in the static state.

Regarding egocentric distance perception, the problem that first needs to be solved is how to measure it; in other words, it is about the methods for egocentric distance perception (Loomis and Philbeck, 2008). The unique characteristics of egocentric distance perception determine the importance of measurement methods. Individuals can perceive the distance accurately, but they are disabled to speak the distance through introspection. Such as, people can easily and accurately take up a cup, but it is hard to say how far away it is. Some argued that egocentric distance perception was an automatic or unconscious perceptual procedure (Blake and Sekuler, 2006). Because of this, verbal report only partially reflects egocentric distance perception and lacks precision (Philbeck and Loomis, 1997). An optimal method for egocentric distance perception is action, such as walking to the target or touching the target (Philbeck and Loomis, 1997).

Nevertheless, researchers also need different methods to choose from because of the diversity of distance ranges. Cutting (1995) divided the space around individuals into the private space (0–2 m), the action space (2–30 m), and the vista space (>30 m). The visual system utilizes the binocular parallax to estimate egocentric distance in the private space. Humans can touch objects directly with accuracy. When egocentric distance is beyond 2 m, namely in the action space or in the vista space, it is difficult to show the perceived distance by hand (Bufacchi and Iannetti, 2018). This suggests that researchers need to find other ways to measure egocentric distance perception accurately.

In sum, a systematic understanding of the similarities and differences between the measurement methods is an important guarantee for further exploration of egocentric distance perception. In the action space (2–30 m) and the vista space (>30 m), the researchers measured the estimated distance in the static state by a large number of methods, such as successive equal-appearing intervals distance judgment measurement (Gilinsky, 1951; Ooi and He, 2007), verbal report (Philbeck and Loomis, 1997), perceptual distance matching (Wu et al., 2007a), blind walking (Thomson, 1983), blind-walking gesture (Ooi et al., 2001), blindfolded throwing (Eby and Loomis, 1987), blind rope pulling (Philbeck et al., 2010; Bian and Andersen, 2013), triangulation-by-pointing (Fukusima et al., 1997), and triangulation-by-walking (He et al., 2004). Studies showed that these methods could measure relatively pure perceived distance. The relationship between the measured distance and the practical distance fits the linear function, and there is also a significant positive correlation between the measurement results of different methods (Loomis et al., 1992). However, there are also significant differences in the measurement results between different methods.^1^ The differences also exist in the core logic of measurement, the scope of application, the advantages, the disadvantages, and the precision of the methods (Hutchison and Loomis, 2006; Bian and Andersen, 2013). Thus, our paper according to the type of action divides the methods for egocentric distance into Perceptual Method (not relying on action), Direct Action Method (relying on action), and Indirect Action Method (relying on indirect action). At the same time, the core logic, the measurement procedure, the scope of application, the advantages and the disadvantages of the methods are summarized, to provide a reference for the researchers in the field of spatial perception.

## 2. Perceptual method

Early researchers believed that the egocentric distance could be realized or spoken by verbal reporting or comparing (Loomis et al., 1992). This is a simple and direct way to measure. In this paper, we name the kind of methods ‘Perceptual Method’. It is an early kind of method used in the research of egocentric distance perception, which has the following three characteristics. Firstly, in the process of estimating the distance, it is no need for observers to move. Secondly, judged or reported distance by observers means their perception. Finally, when observers are reporting, there is still continuous visual input. This kind of method involves Successive equal-appearing intervals of distance judgment measurement, Verbal report, and Perceptual distance matching.

### 2.1. Successive equal-appearing intervals of distance judgment measurement

**Figure 1:**
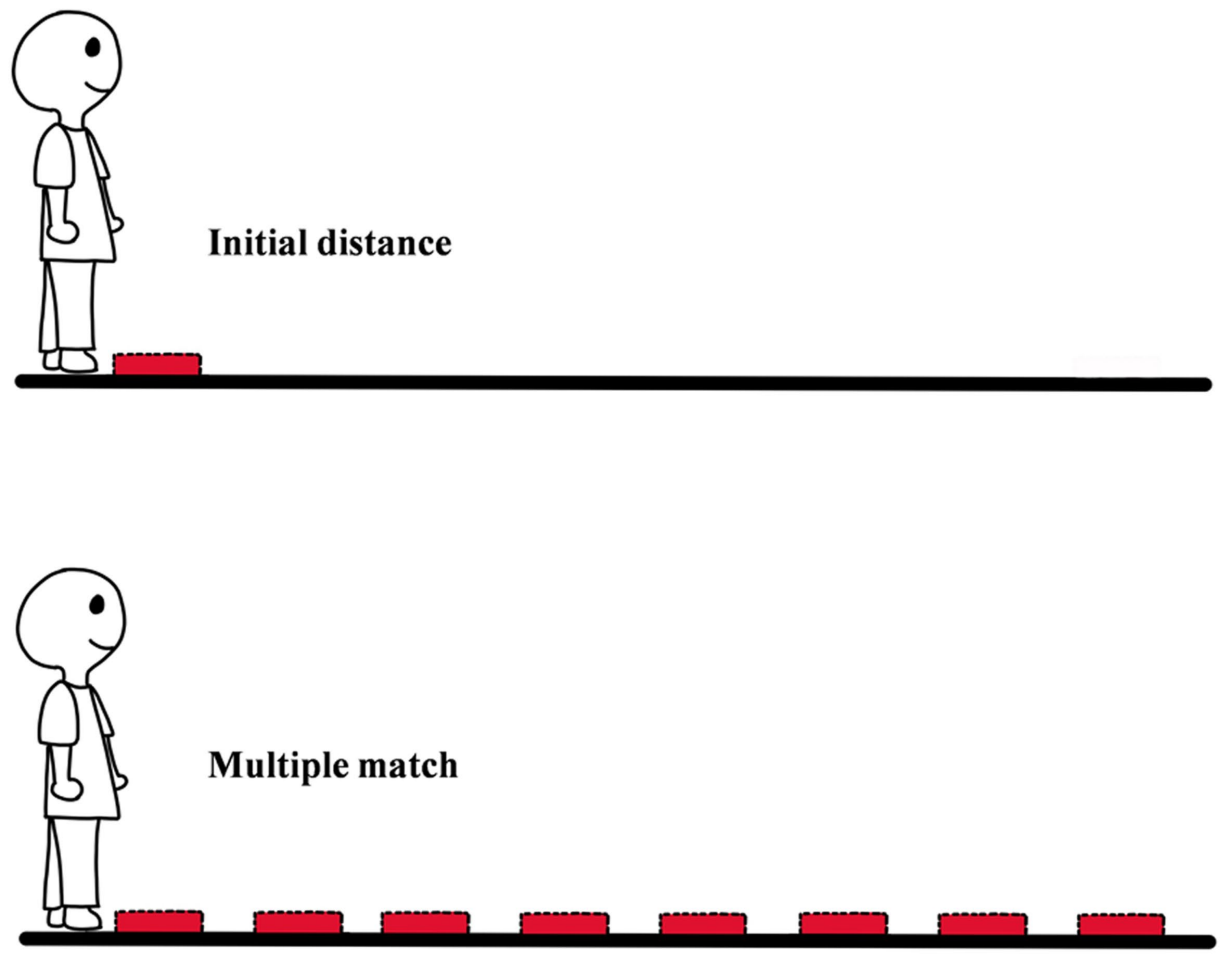
The diagram of successive equal-appearing intervals distance judgment measurement.

An important task of psychology is to describe the relationship between physical quantity and psychological quantity. Gilinsky (1951) developed successive equal-appearing intervals of distance judgment measurement to establish a functional relationship between the physical quantity and the psychological quantity of egocentric distance (Gilinsky, 1951; Ooi and He, 2007). In the experiment of Gilinsky (1951), the observer needed to instruct an experimenter to adjust the distance between a horizontal rod stick and a pointer stick on the ground until the distance equated to 1 foot in the memory of the observer. Throughout the experiment, the observer was required to instruct the experimenter to adjust the intervals from near to far on the ground multiple times (Figure 1).

The following three points are the core logic of the method: (1) Distance in the observer’s memory is a psychological quantity; (2) The memory of distance can be extracted; (3) The psychological quantity of egocentric distance can be added but not multiplied, namely in terms of the psychological quantity, 2 feet is the sum of the 0 to 1 foot and the 1 to 2 feet, not twice the 0 to 1 foot. In practical use, if the psychological quantity of a standard distance (e.g., 1 foot) can be adjusted continuously at equal intervals on the ground, the psychological quantity of any distance can be calculated. Using the method, Gilinsky (1951) found that the farther the location was, the longer 1 foot in the observer’s memory was. Egocentric distance (the physical quantity) and estimated distance can fit a specific equation, namely Gilinsky Equation.


$$d=\frac{DA}{D+A}$$

*d* is the psychological quantity of egocentric distance; *D* is the physical quantity of egocentric distance; *A* is a constant, representing the scale value of the distance. The value of *A* depends on the observer and depth cues in the current environment.

Successive Equal-appearing Intervals Distance Judgment is one of the earliest experimental methods to study egocentric distance perception by psychophysical method (Gilinsky, 1951; Ooi and He, 2007). The results of the method not only showed that there was a correspondence between physical quantity and psychological quantity but also established the function between the two (Gilinsky Equation). However, there are two deficiencies in the method. In the first place, the psychological quantity of the first 1 foot relies on the observer’s memory, and the individual difference will affect the result. Given the problem, Ooi and He (2007) revised the method. In their experiment, researchers first placed a graduated tape measure in front of the observer. Then they would ask the observer to look at the graduated position of a distance, such as 2 feet, and to memorize the distance from the tote to the position as a reference for the subsequent distance (Ooi and He, 2007). In the second place, starting from the second interval, the distance in the method is the exocentric distance instead of the egocentric distance. This leads to the calculated psychological quantity of 1 foot involving both egocentric distance and exocentric distance; for example, estimated 3 feet = egocentric distance (0 to 1 foot) + exocentric distance (1 to 2 feet) + exocentric distance (2 to 3 feet). In addition, the longer the distance, the greater the effect of exocentric distance. This flaw may be one of the reasons why the distance compression in the Gilinsky Equation is much larger than in other studies (Loomis et al., 1996). For example, the studies that used blind walking found that observers could accurately perceive egocentric distance out to 25 meters. However, when using successive equal-appearing intervals distance judgment, the observer could only perceive the distance accurately out to 5 meters. Because of this, in recent studies, researchers little used the method. Nevertheless, the Gilinsky equation revealed an important and still closely watched phenomenon in the field of distance perception and spatial perception–perceptual compression of distance. Therefore, researchers still often mentioned the method in recent papers.

### 2.2. Verbal report

**Figure 2:**
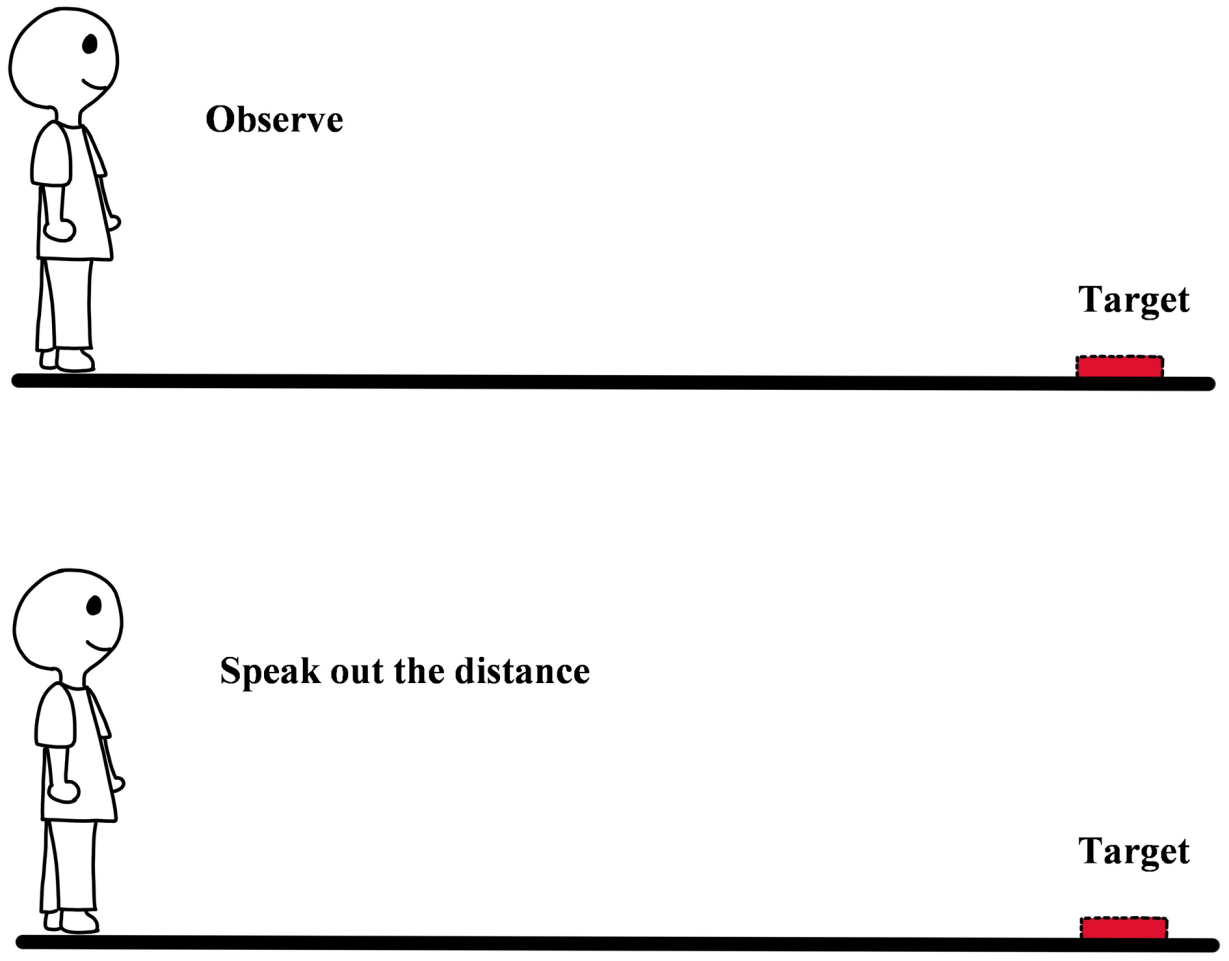
The diagram of verbal reporting.

Verbal report is that when an observer estimates egocentric distance, he/she gives a quantitative estimate of the distance in specific units, and in some studies, it was called “Magnitude Estimation” (Loomis et al., 1992; Philbeck and Loomis, 1997; Loomis and Philbeck, 2008). Specifically, the procedure of verbal report is relatively simple. The first is that observer looks at the target on the ground and tries to estimate the distance between himself/herself and the target. Secondly, the observer needs to report the result of estimating in a specific unit (e.g., feet, inches, yards, meters, centimeters, etc.) and the estimation can be regarded as the psychological quantity of egocentric distance (see Figure 2; Wu et al., 2007b). Verbal report is also an early method that was used to measure egocentric distance perception. The core logic of the method is that the perception of the observer is at the level of consciousness, and it can be spoken out. The distance reported by the observers means their naive cognition of distance. Because in daily life, people often use oral ways to express their cognitive results.

Compared to the successive equal-appearing intervals of distance judgment measurement, verbal report is a relatively simple and direct method that is not disturbed by exocentric distance perception (Wu et al., 2007b). In addition, verbal report can be used to measure distance perception under different conditions in a short time. Due to the advantages above, the method has been widely used in the domain of ground representation, spatial training, traffic safety, and others (Cavallo et al., 2001; Wu et al., 2007b; Bernhard et al., 2021). However, the method has also been challenged on two points over the years. The first is subjective cognitive correction. Adults are generally aware of how their perception varies with distance, such as the principle “the farther the distance is, the smaller the object is.”. Therefore, when adults report the estimation of distance, they may intentionally or unintentionally correct the result. To solve the problem, some researchers have tried to make observers report apparent distance instead of objective distance that was consciously corrected (Loomis et al., 1992). However, in the method observer has to measure the apparent distance in a unit. It is difficult to eliminate cognitive correction from the measurement procedure. Furthermore, the apparent distance is variable, which can also make the measurements unstable. Second, the core logic of the method is that perception can be spoken out. The logic is not entirely correct. As mentioned above, it is hard to speak out exactly how far away the cup is in daily life. Even though, before reporting people carefully observe the distance, they would also question their answers. It is worth noting that the sense of doubt and uncertainty does not mean that people cannot accurately perceive the distance. For example, a person can easily and accurately pick up a cup even with his/her eyes closed. It means that the perception of people is accurate. Although, like other measurement methods, the perceived distance measured by verbal report is smaller than the physical quantity, the distance reported by observers is significantly smaller than that measured by other methods (e.g., blind walking; Napieralski et al., 2011). At the same, verbal report is less reliable and less stable compared to other methods (Rand et al., 2019). This means that the reported distance is only part of the perceived distance, not all of it.

Because of its convenience and understandability, verbal report is still one of the commonly used methods in physical distance research. Different from the early studies, when researchers recently used verbal report to measure, they would supply other methods to cover the shortage of verbal report.

### 2.3. Perceptual distance matching

**Figure 3:**
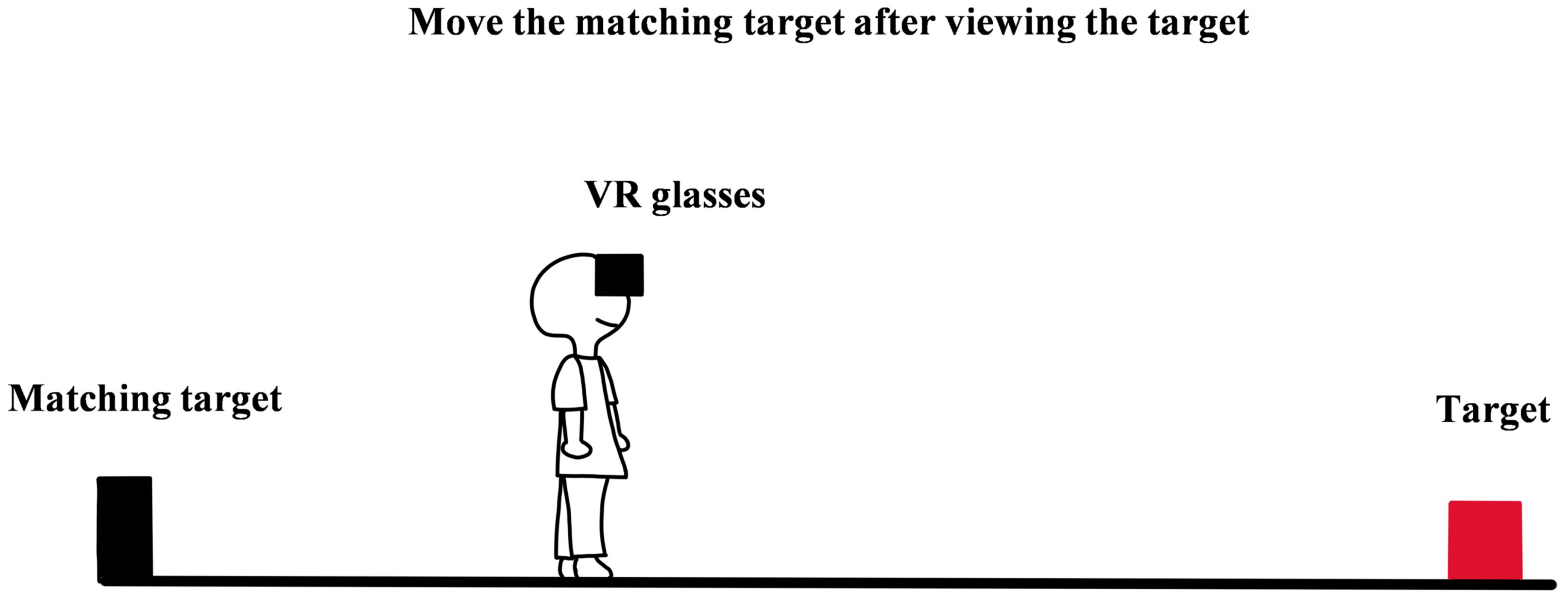
The diagram of perceptual distance matching (in virtual reality).

Perceptual distance matching is the method of showing egocentric distance perception of the observer by moving a matching target (Sinai et al., 1998; Wu et al., 2007a). To be more specific, in an experiment, after the observer views the target and remembers egocentric distance, he/she can turn around by 90°or 180°and instruct the researcher (or use remote control) to adjust the matching target on the ground until it is at the same distance as the target. In the process of adjusting, the observer can turn around multiple times to confirm whether the distance of the test target and the distance of the matching target are equal and adjust the matching target until satisfied without a limit of time (see Figure 3). Finally, the distance between the matching target and the observer can be regarded as the perceived egocentric distance of the target.

The core logic of Perceptual distance matching is setting the matching target. Although the method still assumes that perceived distances are in the individual’s consciousness, the setting of the matching target transforms the perception of an absolute distance into a comparison of two absolute distances. In the comparison, the observer does not have to report the distance in a specific unit and just needs to adjust the location of the matching target to make the two distances look equal. Because of this, the method avoids the bias caused by prior knowledge of distance. To a large extent, the two deficiencies of verbal report, which are cognitive correction and “perception can be spoken out,” can be overcome.

Due to the principle of near is bigger and far is smaller, namely size-distance invariance, the estimation of distance would transform into the estimation of size when the matching target and the target are in the same field of vision. To avoid the effect of size, the experimenter generally would set the matching target at a different angle (usually on the opposite side of the target), so that the observer must turn around to see the matching target. A study showed that there was no significant difference between the matching distance (3.54 ± 0.07 m) and the physical distance (3.66 m; Sinai et al., 1998). This means that the method is effective and accurate.

Perceptual distance matching does not depend on the verbal report or the direct action and has a wide scope of applications. On the one hand, because of the matching target and the target on the different sides, the method is easy to use in the field which is inconvenient to walk, for instance, on the mountain, water, and ground with pits or convex. On the other hand, during matching, the observer does not need to use the absolute or actual units of length, so the method can be used in a context with large systematic errors, and to some extent, the errors can be offset in the process of matching. For example, in virtual reality, the bias of distance perception is large, but perceptual distance matching is still appropriate for the scenario (Wu et al., 2007a; Dong et al., 2021a, b). More importantly, neither the verbal report nor the action-based method cannot be applied in the vista space (>30 m), but perceptual distance matching has no such limitation.

### 2.4. The summary of perceptual method

In short, all the three methods above are the traditional methods in the studies of egocentric distance perception, and the observer expresses the psychological quantity of distance through verbal reporting or distance matching. The advantages of the perceptual method are simple principles, direct measurement, and easy to understand. The disadvantage is that it is difficult to exclude the influence of subjective cognitive factors. When using the method, the observer is always with visual input. Because of this, it is easy to correct the perceptual result. This makes the inherent mechanisms of the three perceptual methods complex, and slight factor variation may lead to a change in the measured distance perception.

## 3. Direct action method

As the understanding of perceptual paradigms deepened, researchers found that the action of body moving could more accurately and directly show egocentric distance, so they developed several measurement methods that are still widely used today. In this paper, the kind of paradigm is called the direct action paradigm, including blind walking, blind walking gesturing, blindfolded throwing, and blind rope pulling. The characteristics of the direct action paradigm are as follows: (1) The observer expresses the psychological quantity of egocentric distance by action rather than verbal reporting, (2) The observer expresses the distance to the target by approaching it in a straight line, which does not involve the change of angle (Thomson, 1983; Eby and Loomis, 1987; Loomis et al., 1992; Fukusima et al., 1997; He et al., 2004; Bian and Andersen, 2013).

### 3.1. Blind walking

**Figure 4:**
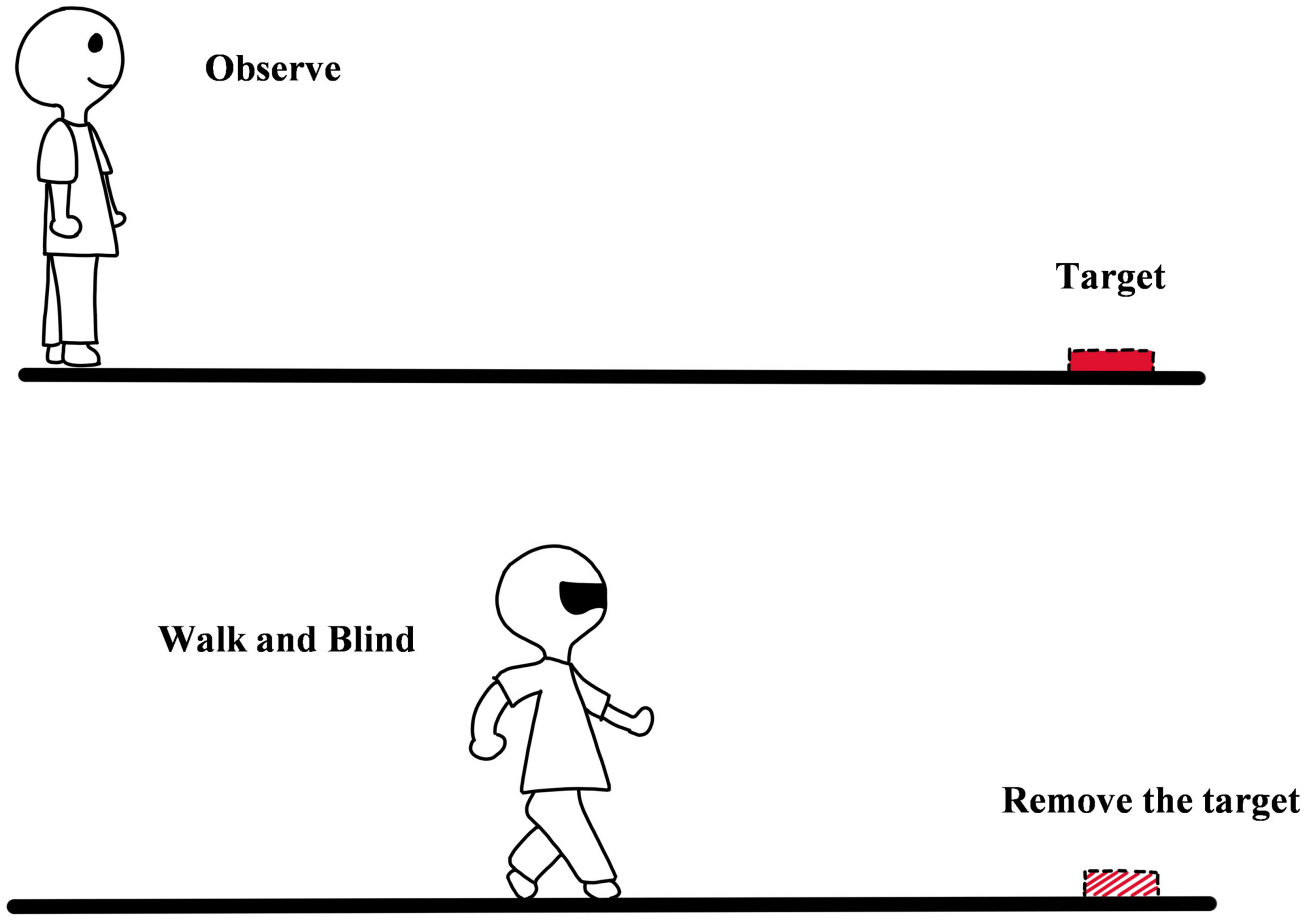
The diagram of blind walking.

Blind walking is widely used in the studies of egocentric distance perception, in which the observer carefully observes and remembers the location of the target and then walks to the location of the target with eyes closed or a blindfold (see Figure 4; Thomson, 1983; Philbeck et al., 2010; Teng et al., 2016; Zhou et al., 2016; Li, 2017; Siegel and Kelly, 2017; Kelly et al., 2018). The method was developed by Thomson (1983). His experiment was carried out in a flat space. The experimenter set up two pathways 6 meters apart on the ground, and the target was placed about 1 meter to the left of the walking path. The task of the observer was to walk toward the target with his/her eyes closed and place his/her foot as close as possible to the center of the target. To avoid flaws of the method, Thomson (1983) did his best to deprive the vision. In his preliminary experiment, he tried a variety of eye masks, but he found the observers could not walk naturally at a reasonable speed. So instead of eye masks, he asked participants to close their eyes to deprive the visual input. Before the formal experiment, participants were generally instructed to practice several times to walk naturally with their eyes closed. In the formal experiment stage, the experimenter would put the target in the specified position and ask the observer to watch the target for 5 s. And then, the observer closed his eyes and walked toward the target. After the observer stopped walking, he/she still had to close eyes and wait for the experimenter to mark where the observer stopped and move the target to the other position for the next trial. At the same time, another experimenter would take the observer back to the starting point still with eyes closed to avoid the observer receiving information about how well he/she had done in the previous trials (i.e., no feedback; Thomson, 1983).

The core logic of blind walking is reflecting perceptual performance by walking. The method involves not only the perceptual process of distance but also the memory of the initial location of the target, proprioception, and spatial updating (Loomis et al., 1996). The method has three advantages: (1) The method does not depend on the consciousness of the observer and is not affected by the subjective cognitive correction. When using the method, it is no need for observers to use any units of length (i.e., meters, feet, etc.) as a reference. They just need to remember the apparent distance. So blind walking can be performed even if the observers do not recognize or remember any units of length. (2) It is more reasonable for observers to show perceived distance by walking. The default unit for walking is the length of the step. As human beings need to walk every day and always measure the distance of objects around them by step, step becomes the most familiar unit of length for humans. (3) Walking with eyes closed avoids the feedback. Compared to the continuous vision input of Perceptual Method, blind walking shields the visual stimulus during walking. Therefore, the method further avoids observers optimizing the perceptual results while walking.

In addition to traditional blind walking, the researchers have developed some variations while keeping the core logic: (1) People can walk blindly on a treadmill instead of on the ground. This task is called blind treadmill walking (Proffitt, 2006; Bossard et al., 2020). (2) Walking can also be done instead of imagining. The experimenter first measured the observer’s conventional walking speed and then asked the observer to imagine walking to the target with his eyes closed. After recording the walking time, egocentric distance perception could be obtained by multiplying the walking speed. The task is called timed imagined walking (Plumert et al., 2005).

Blind walking is also restricted by several factors. The first is walking time. Thomson (1983) found that the duration of the test to finish the task would affect their performance. If observers finished the task within 8 s, they were able to accurately perceive egocentric distance within a range of 21 meters. If not, the accuracy of perception would decrease rapidly. This may be due to the limited holding time of the motion program (Thomson, 1983). The second is the need for space. Blind walking has high requirements on the space of the experiment field. There is plenty of space outside, but it is easily disturbed by bad weather; Indoor can eliminate the influence of the weather, but it is easy to collide with the objects around because of the narrow corridor. Some tried to use blind walking in virtual reality, but the limitation of devices could result in insufficient measuring range or a large number of system errors (Philbeck et al., 2010; Li, 2017; Siegel and Kelly, 2017; Kelly et al., 2018). The Final is the task load and the time limit of the whole experiment. Blind walking takes time and effort. It is difficult for the aged and the brain injury patient to finish the task (Bian and Andersen, 2013; Wallin et al., 2017), and in extreme cases, participants can get hurt, so blind walking may not be the best choice when studying special populations (elders or patients).

### 3.2. Blind-walking gesturing

**Figure 5:**
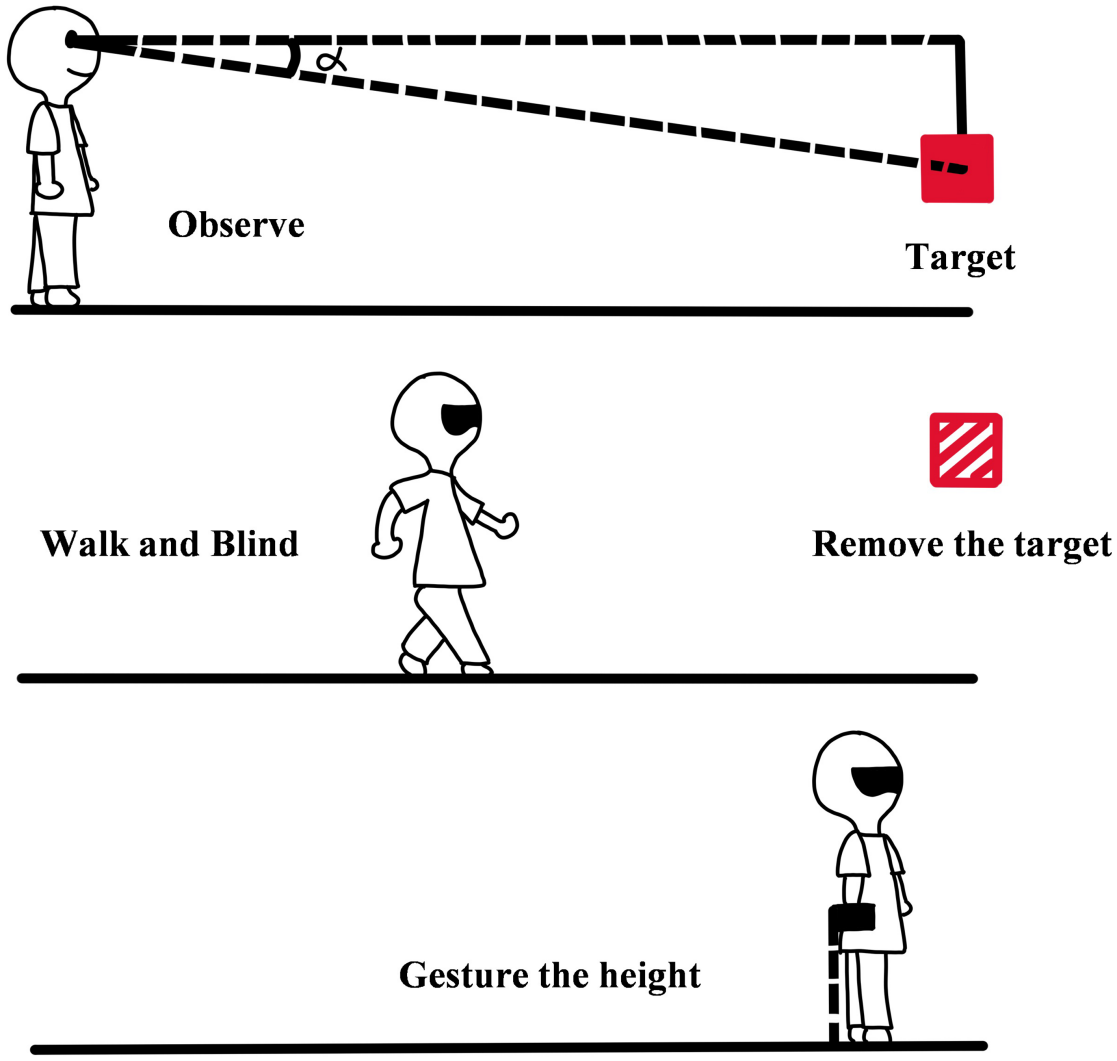
The diagram of blind walking gesturing.

Blind-walking gesturing refers to the method in which observers first view the target and judge distance and height, then walk blinding according to the distance of the target in memory, and show the perceived height of the target with gesturing after reaching the location of the target (see Figure 5; Ooi et al., 2001; Chen et al., 2016). Blind-walking gesturing is a classical variant of blind walking, and the gesturing height is added to represent the perceived vertical height of the target (Ooi et al., 2006). The specific procedure of the method is as follows.

There are two stages in the method, including the perceiving stage and the reacting stage. In the perceiving stage, the observer is asked to observe the target and remember its egocentric distance. After finishing the observation, the observer should wear a blindfold, and this means that the perceiving stage is over. And then entering the reacting stage. At the time, the experimenter will quickly take the target away and instruct the observer to walk to the remembered location of the target. After the observer arrives, he/she will gesture the height of the target with hand. There is no feedback in each trial. The method can not only measure the accuracy of distance judgment but also calculate the perceived direction of the target by gesturing height. The indicator of the perceived direction in the method is the angular declination below the horizon, which is represented by *α*. The calculation formula of *α* is *α* = arctan [(*h_eye_*–*h*)/*d*] × 180 / π (*h_eye_* is the eye height of the observer, *h* is the actual height of the target, *d* is the actual distance from the target to the observer). The psychological quantity of *α* is *α, ‘,* and the calculation formula of it is *α’* = arctan [(*h_eye_*–*h_g_*) / *d_w_*] × 180/π (*h_g_* is the height of the observer gesturing, *d_w_* is the horizontal distance of the observer walking blind). By comparing *α* with *α’*, the accuracy of the observer’s direction judgment can be obtained. Different from blind walking, the distance from the eye to the target is used as the indicator of distance estimation in the method. The calculation formula of the indicator is *d_eye-to-target_* = √ [*d_w_*^2^ + (*h_eye_*–*h_g_*)^2^] (*d_eye-to-target_* is the distance from the observer’s eyes to the target, the meanings of other letters are consistent with the formula of *α* and *α’* (Ooi et al., 2001; Chen et al., 2016).

Blind-walking gesturing is added to the measurement of the target height based on blind walking; therefore, the research scope is no longer limited to the distance judgment on the ground and can be extended to the direction and height of the suspended target. Some found that in the dark, the observer would underestimate egocentric distance, but they could accurately perceive the direction of the target (Thomson, 1983; Ooi et al., 2001, 2006).

The innovation of the method is that it extends the perceptual representation limited in the two-dimensional plane to the three-dimensional space and integrates the distance and the direction judgment into a method. It enables researchers to obtain more data in a study, which not only improves the research efficiency but also enables researchers to study spatial perception more comprehensively.

### 3.3. Blindfolded throwing

**Figure 6:**
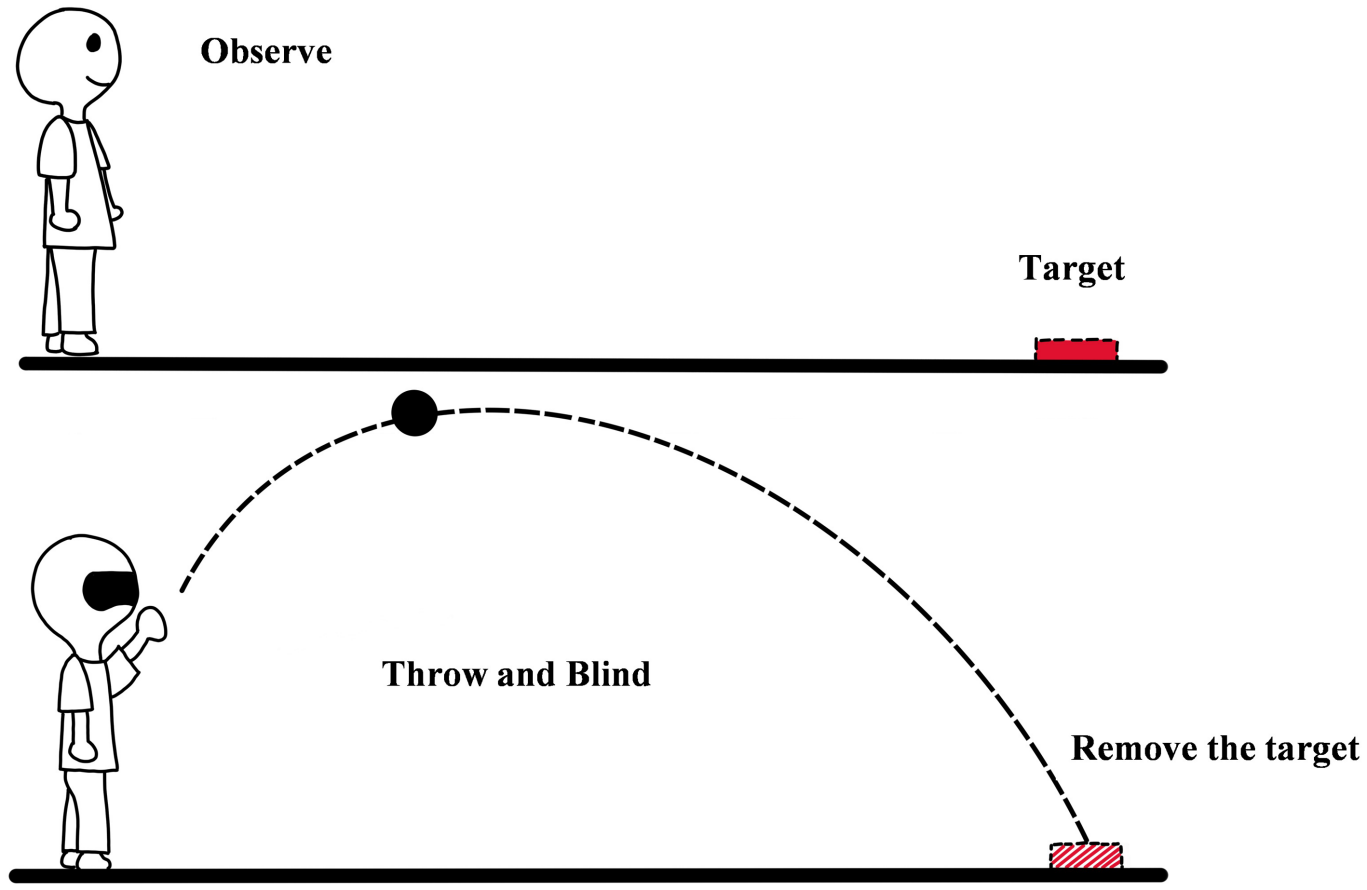
The diagram of blindfolded throwing.

Blindfolded throwing refers to the method in which the observer throws an object (such as a bean bag) with eyes closed or covered after observing the location of a target (see Figure 6; Eby and Loomis, 1987; He et al., 2004; Peer and Ponto, 2016). The method is derived from the study of Eby and Loomis (1987). In the method, the observer first constructs the representation of egocentric distance to the target. And then, he/she programs an action based on the representation. Finally, the observer acts (e.g., throwing a bean bag) with his eyes closed to show the perceived egocentric distance (Eby and Loomis, 1987). The method is similar to blind walking. The throwing distance can be regarded as the egocentric distance perception of the observer, and the performance of throwing is equal to the accuracy of egocentric distance perception. In the field of distance perception, the method is not commonly used, but sometimes, it would be used to explore interesting theoretical questions. For example, He et al. (2004) used the method to verify the sequential-surface-integration- process (SSIP) hypothesis (He et al., 2004). In the frame of SSIP, an obstacle on the ground between the observer and the target would make the observer underestimate the egocentric distance to the target. To verify the hypothesis, He et al. (2004) designed an experiment with two conditions. One condition was with an obstacle on the ground, and another was without an obstacle on the ground. The tasks in the two conditions were the same. The observer needed to view the target and judge egocentric distance. And then he/she threw a bean bag to the location of the target. The result showed that compared with the non-obstacle condition, the throwing distance of the observers under the obstacle condition was shorter. This means that SSIP is true (He et al., 2004).

The limitation of the method is obvious. The accuracy of it is lower than that of blind walking, and the performance of throwing is easy to be affected by the ability of the individual and practice. Eby and Loomis (1987) found that their participants were able to accurately throw with eyes closed, even when the distance was 15 m, but in the experiment of He et al. (2004), their participants could not do the same things. By comparison, the participants of Eby and Loomis (1987) had stronger athletic ability and stronger throwing ability than those of He et al. (2004). This means that the motion of the observer needs to be balanced in advance when using the blindfolded throwing. In addition, He et al. (2004) also found that the feedback in the practice stage was beneficial to the performance of the participants. The foundation is in line with Eby and Loomis (1987), so whether the feedback is given is also an important factor that should be under consideration in the method.

### 3.4. Blind rope pulling

**Figure 7:**
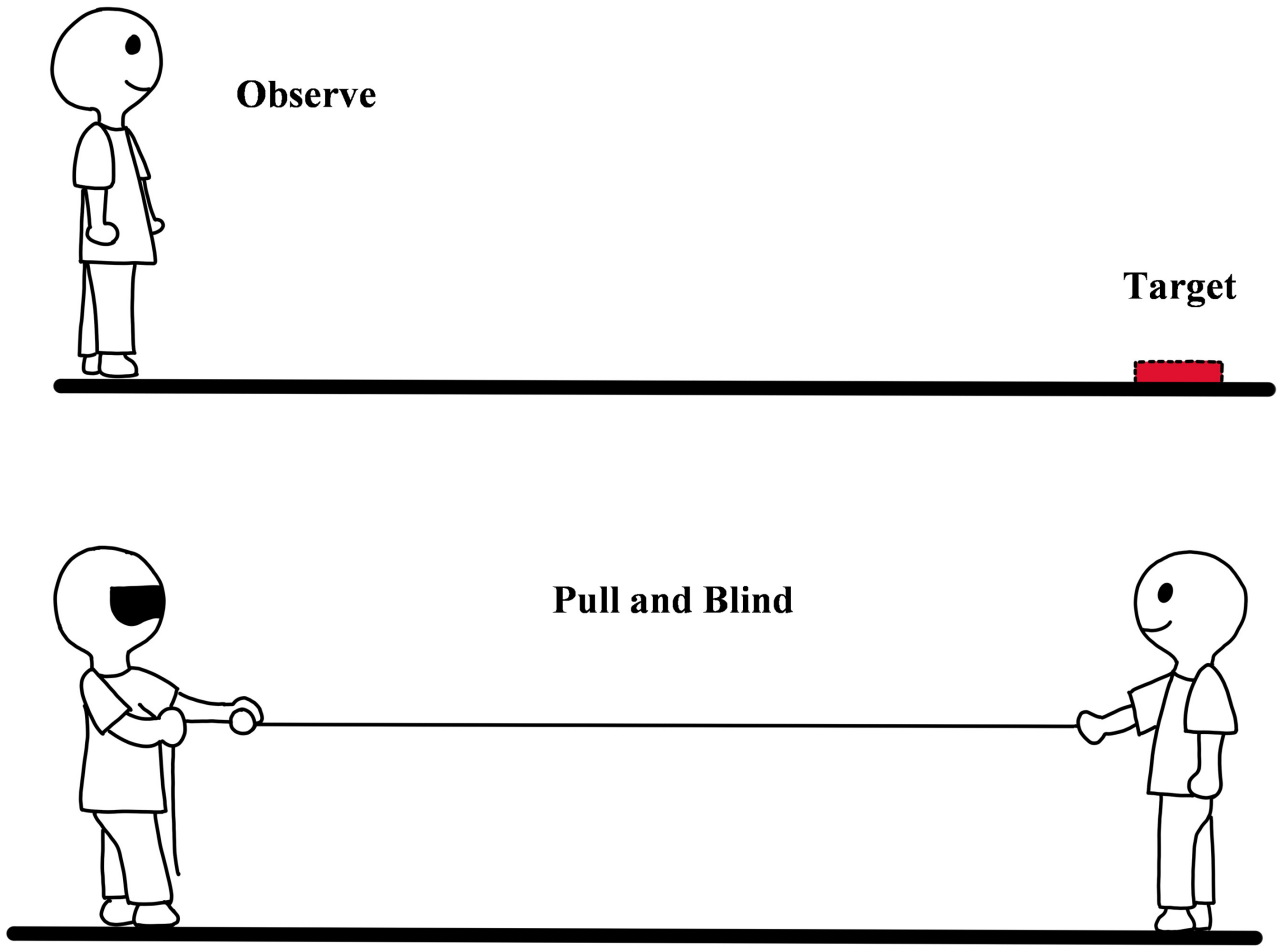
The diagram of blind rope pulling.

Blind rope pulling refers to the method in which after observing the target, an individual imagines that the target is tied to a rope and pulls the rope with eyes closed until the imagined target is pulled to the hand (see Figure 7; Philbeck et al., 2010; Bian and Andersen, 2013). In some cases, the rope can be used instead of the tape measure to measure easier. Philbeck et al. (2010) developed blind rope pulling based on blind walking. The procedure of measurement is as follows.

During measurement, the observer first practiced pulling the rope and formed knowledge of the speed and the length of the rope. And then, he/she observed the target and its location until being ready. After observation, he/she needed to close the eyes and prepare to pull the rope. During pulling, an experimenter would stand at the location of the target and place a tape (easier to measure the distance) around his waist. The experimenter should remember the tape measure scale (point A) where the hands are, and the zero which is the end of the tape measure was held by the observer. After the experimenter gave the signal, the participant started to pull the tape measure until the distance pulled was equal to the perceived egocentric distance. During the stage, the experimenter let the tape measure pass freely. When the participant reported that the pulling was over, the experimenter would record the scale (point B) of the tape measure in hand. The perceived egocentric distance could be measured by B-A. To get more data, the time of pulling and the number of pulling times could also be recorded (Philbeck et al., 2010). There is a high correlation between the results of blind rope pulling, real egocentric distance, the results of perceptual method, and blind walking, which indicates that the method could measure egocentric distance perception (Philbeck et al., 2010; Bian and Andersen, 2013).

Eye closing and pulling rope are the two core settings of the method. Before the development of the method, the researchers had used another two methods. One is ‘pass the rope through the subject’s hands without arm movement’ (Mershon et al., 1977) and the other is ‘pull the rope with eyes opened’(Hagen and Teghtsoonian, 1981), but these methods lack precision. The core logic of blind rope pulling is similar to blind walking. The former uses the familiar arm length as a reference, while the latter uses the familiar step length as a reference, both of which reflect humans using the body to perceive the outside world. Before the formal experiment, observers would be asked to perform rope-pulling exercises with visual stimulation, which could effectively improve the accuracy of the task. And observers can calibrate their movements by visual rope, consciously adjust their movements, and reduce the errors caused by improper action. Compared to blind walking, the method is also easy to be affected by the design of the experiment. In a complex experimental design, its performance was different from that of blind walking (Philbeck et al., 2010). There are two distinct advantages of blind rope pulling over the blind walk: (1) The method has lower requirements on the environment, and the scope of application is wider. It can be used not only on flat land, but also on the sea, lakes, and even on the edge of cliffs. Due to the small requirement of space for pulling and the experimenter who holds the rope being not necessary, even in a narrow space, the method still can be applied. It is worth noting that the method can be used to study not only the distance perception all around but also the distance perception above and below the observers. (2) Since the method does not require the ability of walking, it is more suitable for studying the distance perception of the elder and the brain injury patient.

### 3.5. The summary of direct action method

The direct action method was most frequently used in recurrent studies. The similarity of the direct action method is that the subjects show the perceived egocentric distance directly through action, relying on the perceptual representation or maintained memory after visual deprivation (Bian and Andersen, 2013). Some believed that these tasks reflect the ‘post perception rather than distance perception (Proffitt, 2006; Hajnal et al., 2016). However, other researchers have empirically proved that even when the stimulus has disappeared, the observer still could keep the memory of the spatial image and the accuracy was greater than in other methods (Hutchison and Loomis, 2006). The direct action method is limited by three factors: 1) The initial visual coding of the target position, (2) Perception and integration of body movements by the observer over time, and (3) Motion control and response selection. The perceptual errors of observers often come from the perception and integration of the motion and the control and the selection of the motion. Since the direct action method involves the body movement of the subject, it takes a long time for each trial, and the subject and the experimenter have to spend a lot of time and energy.

## 4. Indirect action method

In the perceptual method and the direct action method, the target is in a straight line, so it can only prove that human beings can accurately perceive egocentric distance in a straight line. If observers have accurately perceived egocentric distance, they should be able to accurately represent the position of the target in more complex behavioral tasks such as multiple locations in space (Loomis et al., 1992). In the paper, the methods that require the observers to follow a curve (not a straight line) while walking or pointing toward the target with their eyes closed are called the Indirect Action Method, including triangulation-by-pointing and triangulation-by-walking. The core logic of the method is that after the stage of perceiving, observers do not directly walk towards the target, but walk along a path with a certain angle to the target. The method can help to confirm whether the representation of egocentric distance is limited by the straight line between the target and the observer.

### 4.1. Triangulation-by-pointing

**Figure 8:**
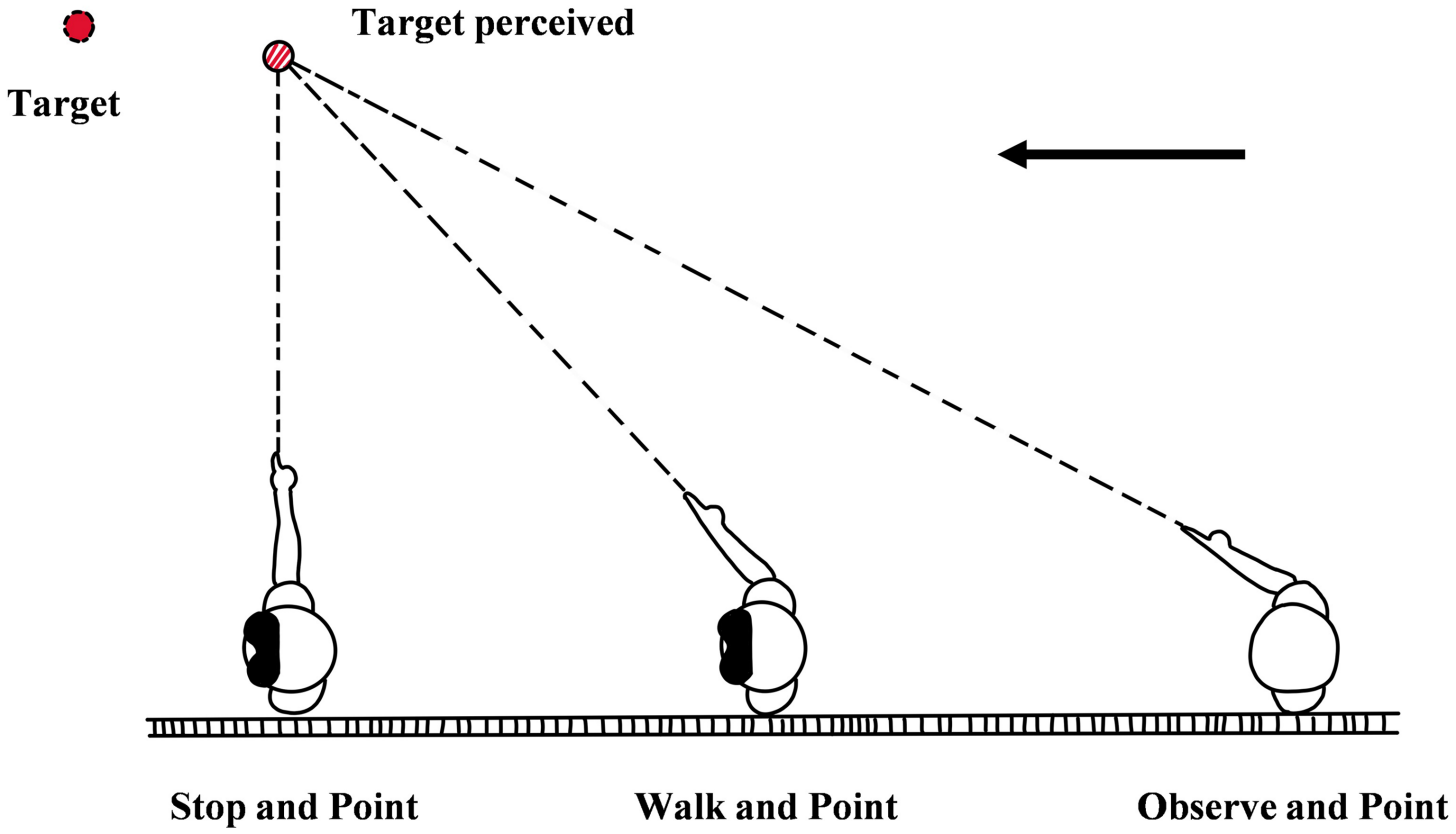
The diagram of triangulation-by-pointing [referenced and modified from Loomis et al., 1992].

Triangulation-by-pointing is also called continuous pointing. It refers to the measurement that after memorizing the location of the object, the individual closes his/her eyes, faces the object, and walks forward while continuously pointing in the direction of the remembered object (see Figure 8; Loomis et al., 1992, 1996; Fukusima et al., 1997; Burkitt et al., 2020). In the method, after viewing the target at the start point, the observer needs to stand flanker to the target. Then he/she must point with a finger in the direction of the target, and the experimenter will record the angle of the finger with an instrument. After that, the observer will close eyes and start to walk forward with pointing to the target. When the experimenter gives a sign, the observer will stop and the experimenter will record the angle of pointing. In addition to the way above, the experimenters can also record the direction of pointing, so that they can affirm whether the observer can represent the location of the target in different places. The perceptual distance in the method is indicated distance which can be calculated by some physical indicators, such as walking distance, the direction of pointing, and the distance between the target and the observer. To verify the effectiveness of triangulation-by-pointing, except for the closing eyes condition (without vision while walking), the experimenters set another condition-opening eye condition (with vision input while walking) as a comparison; namely, the observer needs to keep eyes open while walking and pointing. The result shows that even in the condition without vision, the observer still can accurately perceive the distance up to 5.7 m. This means that the ability of human beings to perceive distance is not limited to the straight lines (Loomis et al., 1992, 1996; Loomis and Knapp, 2003), and the hypothesis that humans can accurately perceive egocentric distance is true (Fukusima et al., 1997).

The core logic of the method is that the observer does not need to walk directly to the target. Triangulation-by-pointing is similar to the situation in daily life in which an individual can bypass multiple obstacles to reach the bed in a dark room with the light just turned off. The experimental results of the method and the actual life experience both show that human beings can accurately perceive egocentric distance. The action in the method is similar to that in the directed action method, and they can both be classified as visually directed action. Due to this, it has the same advantages as the direct action method. Besides, the other advantages are as follows: (1) In the method, the position of the body, the relative position between the body and the target, and the pointing position keep updating, so the method involves rich psychological processes, such as path integration (Loomis and Knapp, 2003). (2) The method does not depend on the completeness of the path. Therefore, the method is suited to apply to an environment that involves puddles, water, and other obstacles which are not conducive to walking. (3) This method can be used in the study of egocentric distance perception involving location updating, so the researchers can investigate the observer’s perception of the location of the object at any time, and then explore the observer’s perception of egocentric distance in the whole space (rather than the narrow space between the observer and the object).

The limitation of the method is the small measurement scope. In other words, as the target becomes further away, the accuracy of measurement decreases significantly. Loomis et al. (1992) found that if the range of distance was beyond 5.7 m, the accuracy of the method would decrease rapidly (Loomis et al., 1992). Although the accurate distance range could be increased to 15 m by appending the walking distance of observers (Fukusima et al., 1997), the accuracy of the method was still lower than that of the direct action paradigm, such as blind walking (He et al., 2004; Ooi and He, 2007). In addition, the method has not overcome the methodological shortcomings of the perceptual method or the direct action method and the indicator of the method is hard to calculate, so this method is rarely used in current studies.

### 4.2. Triangulation-by-walking

**Figure 9:**
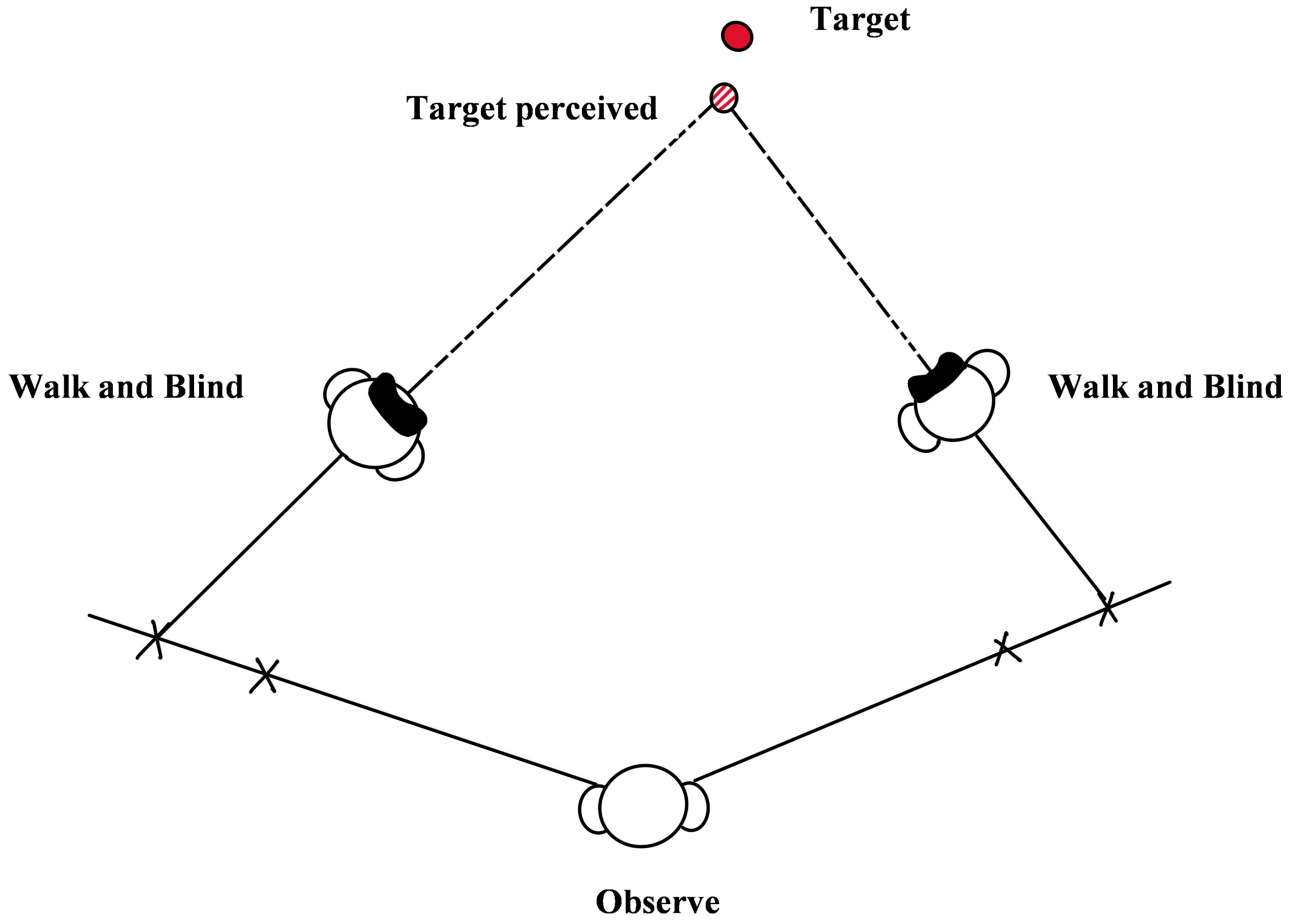
The diagram of triangulation-by-walking [referenced and modified from Fukusima et al., 1997].

Triangulation-by-walking refers to a method in which the observer, after remembering the position of the target, closes his/her eyes and walks along an oblique pathway to the target. After hearing the experimenter’s command to stop, the observer immediately stops and turns around to face the direction of the perceived target, and continues to walk (see Figure 9; Loomis et al., 1996; Fukusima et al., 1997; He et al., 2004; Li, 2017). Loomis et al. (1996) developed the measurement method and in the paper of Fukusima et al. (1997), they introduced the method in detail, involving core logic, measurement process, advantages, and disadvantages (Fukusima et al., 1997). As shown in Figure 9, in the method, the experimenter set up two oblique pathways of the same length on both sides of the starting point. The angle between the two pathways and the horizontal line was equal and the turning point was 4 meters and 6 meters. In each trial, the observer needed to stand at the starting point to observe the target, and after observing over, turn left or turn right to face a random pathway. And then, the observer needed to close eyes and walk along a straight-line slanting to the target. When the observer arrived at the turning point (4 m or 6 m), the experimenter would instruct him/her to turn around to the location of the target in mind with eyes closed. After turning around, the observer still needed to walk forward and try to arrive at the location of the target. When the observer stopped walking, the experimenter would record the direction of walking and then take the observer back to the starting point. Subsequently, the observer needed to go the other way and do the same things as in the first pathway. After the finishing of a trial, the experiment could use the directions of starting point, turning point, and waking pathway after turning around to construct the terminal courses of the observer in the two pathways. The location of the target and the perceived distance can be calculated by the trigonometric function and the terminal courses. It is noted that there is no limitation in the number of oblique pathways, the position of turning, and the walking distance after turning.

The core logic, the advantages, and the disadvantages of triangulation-by-pointing and triangulation-by-walking are roughly the same. The difference between the two methods is that body turning is closer to human daily life and more natural than arm pointing, so the range of accurate measurement of triangulation-by-walking is greater than that of triangulation-by-pointing (Fukusima et al., 1997). And the triangulation-by-walking is suited to the measurement in the whole medium range of distance (2–25 m). However, when using the method in a narrow space, the bias will increase (Klein et al., 2009), so a large physical space is required.

### 4.3. The summary of indirect action method

Verifying the accuracy of distance perception in a more ecological and complex environment is a theoretical contribution of the Indirect Action Method and is also what inspired Loomis and Fukusima to develop the method (Loomis et al., 1992, 1996; Fukusima et al., 1997). From the perspective of cognitive psychology, egocentric distance perception is composed of several sub-psychological processes. The accuracy of egocentric distance perception also indicates the accuracy of the sub-psychological process, including: (1) perceiving accurately the location of the target, (2) perceiving accurately active self-motion, (3) in the process of moving, accurately imaginal updating the location of the target, (4) accurately pointing or walking to the updating position of the target (Loomis et al., 1992; Fukusima et al., 1997). What is noteworthy is that perceiving the location of the target accurately is not equal to constructing the clear spatial representation. The observer can both accurately perceive egocentric distance in the condition with sufficient depth cues and in the condition without insufficient depth cues (Philbeck and Loomis, 1997).

The unique advantage of the method is that the method can be used to study spatial updating. Spatial (or imaginary) updating refers to the mental procedure in which the observer updates the relative position of the target and himself/herself by imagination while moving with eyes closed after remembering the initial position of the target (Loomis and Philbeck, 2008). The direct action method and the indirect action method both involve spatial updating, but the indirect action paradigm is the first method that combines spatial updating with trigonometric functions (Fukusima et al., 1997). In addition, the study on egocentric distance perception was further advanced by the method: the object of study is expanded from egocentric (or one-dimensional) distance to two-dimensional or even three-dimensional space, which improves ecological validity. In other words, the indirect action method measures not only the apparent distance but also the apparent location (Stefanucci and Proffitt, 2009). In the normal condition, when the target is on the ground, the observer can perceive the spatial updating through the reverse change between the angular declination below the horizon and the egocentric distance in the direct action method. However, if the angular declination below the horizon remains unchanged (e.g., the target is vertically upstairs or underfoot), the observer has to perceive the spatial updating by the change of egocentric distance, so in such condition, the indirect action method is more suitable than the direct action method. With the in-depth study, spatial updating has become a unique research topic in the field of spatial perception, and the indirect action method has broad application prospects in the future.

## 5. Discussion

Distance perception is involved in many complex human behaviors, such as driving cars, flying airplanes, playing football, dancing, and so on. Understanding how to measure and similarities and differences between different methods will shed light on the causes of these complex behavior biases and further reduce human error in reality. According to the types of action involved in the measurement methods, nine methods of egocentric distance perception are divided into three categories, and the measurement steps, core logic, applicable scope, advantages and disadvantages of each are introduced, and the advancement of the inherent scientific issues is discussed. What noteworthy is that, although many methods have been used to measure distance perception (see Table 1), the scientific problem of how to measure or probe the psychological quantity of distance has not been satisfactorily solved.

**Table 1:** The summary of advantages and disadvantages of each method for measuring distance perception.

**Methods**	**Advantages**	**Disadvantages**
Verbal report	Intuitively clarify and simple	Influenced by cognitive correction and the observer’s cognition of distance; not suitable to be used alone
Perceptual distance matching	accurate; be applied in the vista space (> 30 m) and used in both real environment and VR environment	Affected by participant’s cognitive factors
Successive Equal-appearing Intervals of Distance Judgment Measurement		Confuse the measurements of exocentric distance and egocentric distance
Blind Walking	Accurate	Take time and effort; easily produce practice and fatigue effects; difficultly applied to far space
Blind-walking gesturing	Integrate the distance and direction judgment, accurate	The same as blind walking
Blindfolded throwing	Simple and time-saving	Participants are required to have a high level of sports ability; inaccurate
Blind rope pulling	Accurate, can be applied to study the elder and patients, even in a narrow space	Participants need to be familiar with rope pulling. The complexity of experimental design affects task performance.
Triangulation-by-pointing	Investigate the rich psychological process; expand the research topic to 3D space	Inaccurate; complex calculation procedure, require larger test space, and takes time and effort
Triangulation-by-walking	More accurate than triangulation-by-pointing, other advantages are the same as Triangulation-by-pointing	The same as Triangulation-by-pointing

It should be noted that the perception methods and action methods (both direct and indirect) may involve different psychological processes. In the perception methods, participants receive continuous visual input, so the measurement may be more relevant to the early perception stage (i.e., perception). However, in the action methods, the visual information is excluded in the measurement stage, which makes the action methods may rely on the distance representation constructed in the observer’s memory (i.e., spatial representation). The relationship between these two psychological processes (completely different or both belong to the perception) still needs to be explored in the future. In the actual study, researchers will choose the appropriate experimental method according to research topics, research objects, and experimental situations. Perceptual distance matching, blind walking, and blind-walking gesturing have higher accuracy; the application scope of perceptual distance matching is large; verbal report is easy to understand and has low space requirement; blindfolded throwing and blind rope pulling are suitable for the larger range of people; triangulation-by-pointing and triangulation-by-walking extend the study object to the whole space. According to the study topic, perceptual distance matching can not only be used to study egocentric distance in the real world but also be used to study egocentric distance and exocentric distance in virtual reality (Loomis et al., 1996; Ooi and He, 2007). For the subjects, all three categories can be applied to normal healthy subjects, but to the special subjects who are difficult to walk, such as elders, stroke patients, and disabled people, the better methods are the perceptual method, blindfolded throwing, and blind rope pulling (Philbeck et al., 2010; Bian and Andersen, 2013). Egocentric distance perception involves three environments: the real world, the virtual world with HMD (head monitor display), and the virtual world with the big screen on the wall. The change in the research environment determines the choice of method. For example, timed imagined walking (a variant of blind walking) is suitable to the environment with the big screen on the wall, blind treadmill walking, blind walking, triangulation-by-pointing, and triangulation-by-walking are suitable to the virtual world with HMD. More recently, a growing number of researchers have combined multiple methods to further improve measurement accuracy based on the convergence principle (Tenhundfeld and Witt, 2017; Rand et al., 2019). Such as combining the verbal report with blind walking (Philbeck and Loomis, 1997), combining blind throwing, triangulation-by-walking, and blind walking to explore the frame of the ground surface and verify the SSIP (He et al., 2004). However, the problems such as what the standard of the convergence principle is and how to deal with the conflicting measurement results under the convergence principle still need to be further solved.

There is no doubt that the method promotes the field of egocentric distance perception, but there are still some common problems that need to be further discussed. The first is the inconsistencies between the results of different methods (Renner et al., 2013). For example, the result of the direct action method is larger than that of verbal report (Philbeck and Loomis, 1997), and the variability of the indirect action method is larger than that of the direct action method (Loomis et al., 1992). The perceptual method is mediated by consciousness and shows the perceptual results through language or matching. The behavioral method (including direct and indirect) bypasses perception and directly shows perceptual results through action. The neural mechanisms of perception and behavioral control are very different: The is mainly controlled by the ventral pathway, while the latter is mainly determined by the dorsal pathway (Loomis and Philbeck, 2008). The difference between methods may reflect the existence of multiple neural structures for distance perception in the human brain. The problem of “what are the differences between methods of egocentric distance perception” may be transformed into “what are the activation conditions of these subcategories”, “what are the mechanisms and rules of subcategories of egocentric distance perception” and so on. The second is about the size of the target. In the experiments of egocentric distance, multiple distance conditions are usually set up simultaneously to investigate the perceptual rule of human beings in the whole space. At this time, the size of the target at each distance becomes a problem that cannot be avoided, because of the covariance of magnitude and distance (Gilinsky, 1951; Loomis and Philbeck, 2008). There are two common approaches to the problem. The one is to keep the physical size of the target; for example, place foam balls 10 cm in diameter at each distance. This method has high ecological validity, but with the increase of the distance, the size of the target in the retina (a kind of depth cue) gradually decreases. When the study problems are about other depth cues (such as binocular parallax or texture gradient), retinal image size (or view angle) will become an interfering variable. Because when the observer views the target, he/she can judge egocentric distance by the size of the retinal image alone and the size perception may replace the distance perception. The two is to keep the retinal image size (or view angle) of the target. Such as, foam balls with a diameter of 1-degree view angle are used at each distance. With the increase of distance, the physical size of the target will gradually increase. Although the method eliminates the interference of view angle, at this time, subjects perceive different objects at different distances. So, it is difficult to compare experimental effects at different distances. Whether keeping physical size or retinal size is a practical problem faced by most spatial perception researchers, the optimal solution has not been formed yet. Researchers can only make choices according to their interested problems. The three is that the methods of static egocentric distance perception measure the perceptual results at the behavioral level and it is difficult to directly measure the perception. Since the internal mechanism of different methods is different, it needs to be further discussed whether the difference comes from internal generative mechanism. The four is that the mechanism and characteristics of spatial perception are mainly discussed in the psychological research, but the relationship between spatial perception and daily life or production still needs to be further strengthened. In the future, psychological studies of space may revolve around the topics of greater application value (e.g., environmental psychology).

With the development of the Metaverse, VR and AR technologies are developing rapidly. The development of these two technologies relies on the research of egocentric distance perception. As far as VR technology is concerned, users should experience a virtual environment that is like our real world to achieve high immersion. For AR technology, users need a high degree of integration of virtual information and the real environment. However, the problem of distance compression has been hindering the VR experience (Kelly et al., 2018) and AR experience (Singh et al., 2018). As a result, VR scenes cannot provide authentic high immersion, and AR content cannot be accurately superimposed on reality. The study of egocentric distance perception can play a role in solving the problem of distance compression. First, it provides a theoretical basis for the cause of distance compression. Second, it provides behavioral indicators for distance compression to measure the degree of compression and verify the effectiveness of solutions. From the perspective of practical needs, how to build a virtual world identical to the real world (Virtual Reality technology) has become an important issue concerned by the government, academia, and even the business community. In the process of product and technology development, developers often use a static method to verify the effect of various factors on egocentric distance perception in VR. For example, the effects of peripheral visual field stimulus presentation, visual field size in virtual space, and other factors on egocentric distance perception were examined using the direct action method (Vaziri et al., 2021), the effects of video card quality, mechanical properties of a head-mounted display, binocular parallax, head scaling and sound scaling on egocentric distance perception was verified by the indirect action method (Choudhary et al., 2021). Although some studies have proved that there is no significant difference in the accuracy of egocentric distance perception between blindfolded throwing and blind walking method in real or virtual environments (Sahm et al., 2005), the construction of realistic depth sense is still the core focus and difficulty in VR (Loomis and Knapp, 2003). The premise to solve the problem is to find or develop a method that can accurately measure depth perception (i.e., egocentric distance perception), that is to find the optimal or most appropriate egocentric distance perception method.

Although this paper focuses on egocentric distance perception in visual modality, auditory distance is a very important research topic. On the one hand, although the current research focuses on egocentric distance in the visual modality, most researchers did not eliminate auditory clues or recruit blind people in the experiments. In other words, there may be an effect of auditory distance perception in the methods mentioned in this paper. It is worth noting that for blind people, auditory distance perception is more important (Voss et al., 2004; Kolarik et al., 2013a,b; Cappagli et al., 2017; Cuturi et al., 2021). In these papers, researchers used different methods (e.g., verbal reporting vs. direct action such as triangle completion) to measure egocentric distance perception. It is also necessary to review this research field. In the future, researchers may focus on this topic.

## Author contributions

BD and XT had the idea for the article. AC and XZ performed the literature research. BD, YS and ZG wrote the first draft of the manuscript. All authors contributed to manuscript revision, read, and approved the submitted version.

## Funding

This work was supported by the National Natural Science Foundation of China (32100841, 32100842, and 71974140), the MOE Project of Humanities and Social Sciences Grant (20YJC190002), the Project of Social Science Foundation of Jiangsu Province (20YJC008), Jiangsu University Philosophy and Social Science Research Project (2019SJA1267), and the Qing Lan Project of Jiangsu Universities.

## Conflict of interest

The authors declare that the research was conducted in the absence of any commercial or financial relationships that could be construed as a potential conflict of interest.

## Publisher’s note

All claims expressed in this article are solely those of the authors and do not necessarily represent those of their affiliated organizations, or those of the publisher, the editors and the reviewers. Any product that may be evaluated in this article, or claim that may be made by its manufacturer, is not guaranteed or endorsed by the publisher.

## Footnotes

^1^There is no contradiction between positive correlation and significant difference between different paradigms. Such as, there is a positive correlation between two groups of data ([1,2,3] and [8,10,12], *r* = 1.00，*p* < 0.001), but there are still significant differences between them (*t*(2) = 13.86，*p* = 0.0052 < 0.01).
